# Supplementary material for: Red-Emitting Carbon Quantum Dots for Biomedical Applications: Synthesis and Purification Issues of the Hydrothermal Approach
Source: Nanomaterials (Basel). 2023 May 13;13(10):1635. doi: 10.3390/nano13101635 (PMC10220864; doi:10.3390/nano13101635)
Supplement: Supplementary file 1 [file nanomaterials-13-01635-s001.zip › nanomaterials-2359843-supplementary.pdf]

Red-Emitting Carbon Quantum Dots for Biomedical Applications: Synthesis and Purification Issues of the Hydrothermal Approach

Barbara La Ferla [1] and Barbara Vercelli [2,*]

[1] Dipartimento di Biotecnologie e di Bioscienze, Università degli Studi di Milano-Bicocca, Piazza della Scienza, 2, 20126 Milano, Italy; barbara.laferla@unimib.it
[2] Istituto di Chimica della Materia Condensata e di Tecnologie per l'Energia, CNR-ICMATE, Via Cozzi, 53, 20125 Milano, Italy
* Correspondence: barbara.vercelli@cnr.it

Contents

Quantum Yields (QY) measurements page S02

Figure S1 page S03

Figure S2 page S04

Figure S3 page S05

Quantum Yields (QY) measurements

QY of the r-CDs was determined by a relative method [32–34]. Rhodamine B (QY = 56% in EtOH) was selected as reference dye for the emission range 580-610 nm.

The QY was calculated according to the following equation:

$$\varphi = \varphi' \times \frac{A'}{I'} \times \frac{I}{A} \times \frac{n^2}{n'^2}$$

where φ is the QY of the testing sample, *I* is the testing sample's integrated emission intensity, *n* is the refractive index (1.33 for water and 1.36 for EtOH), and *A* is the optical density. The superscript " ' " refers to the referenced fluorescence dyes of known QYs. To obtain reliable results, a series of solutions of r-CDs and Rhodamine B were prepared with concentrations adjusted such that the optical absorbance values were between 0-0.1 at 510 nm. The PL spectra were measured, and the PL intensity was integrated. The QYs were determined by comparison of the integrated PL intensity *vs* absorbance curves (refractive index, n, had also be considered).

r-CDs λ = 510 nm (EtOH)

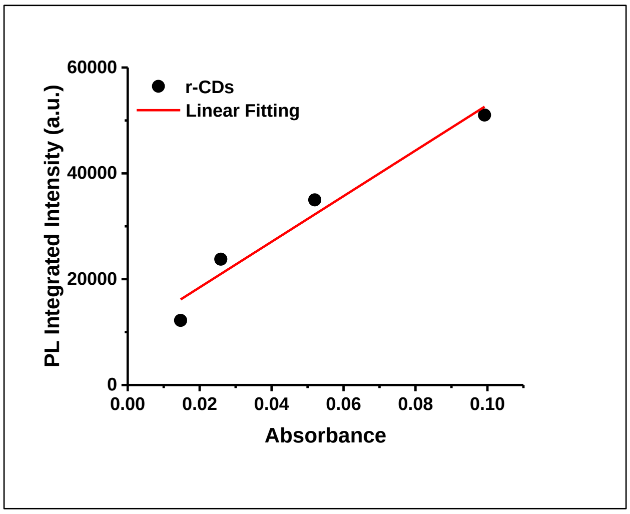

Rhodamine B λ = 510 nm (EtOH)

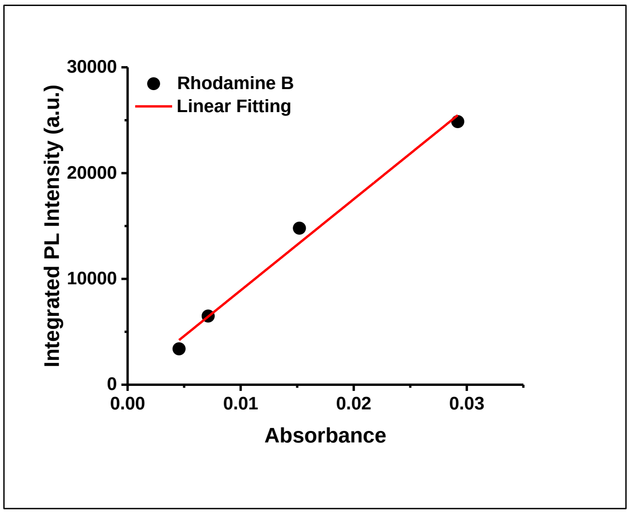

	r-CDs				Rhodamine B			
**Abs**	0.015	0.026	0.052	0.099	0.0046	0.0071	0.0152	0.029
**Integrated PL**	12219	23775	34984	51014	3391	6487	14788	24861
**Slope**	4.3×10^6				8.6×10^6			
**QY**	27%				56%			

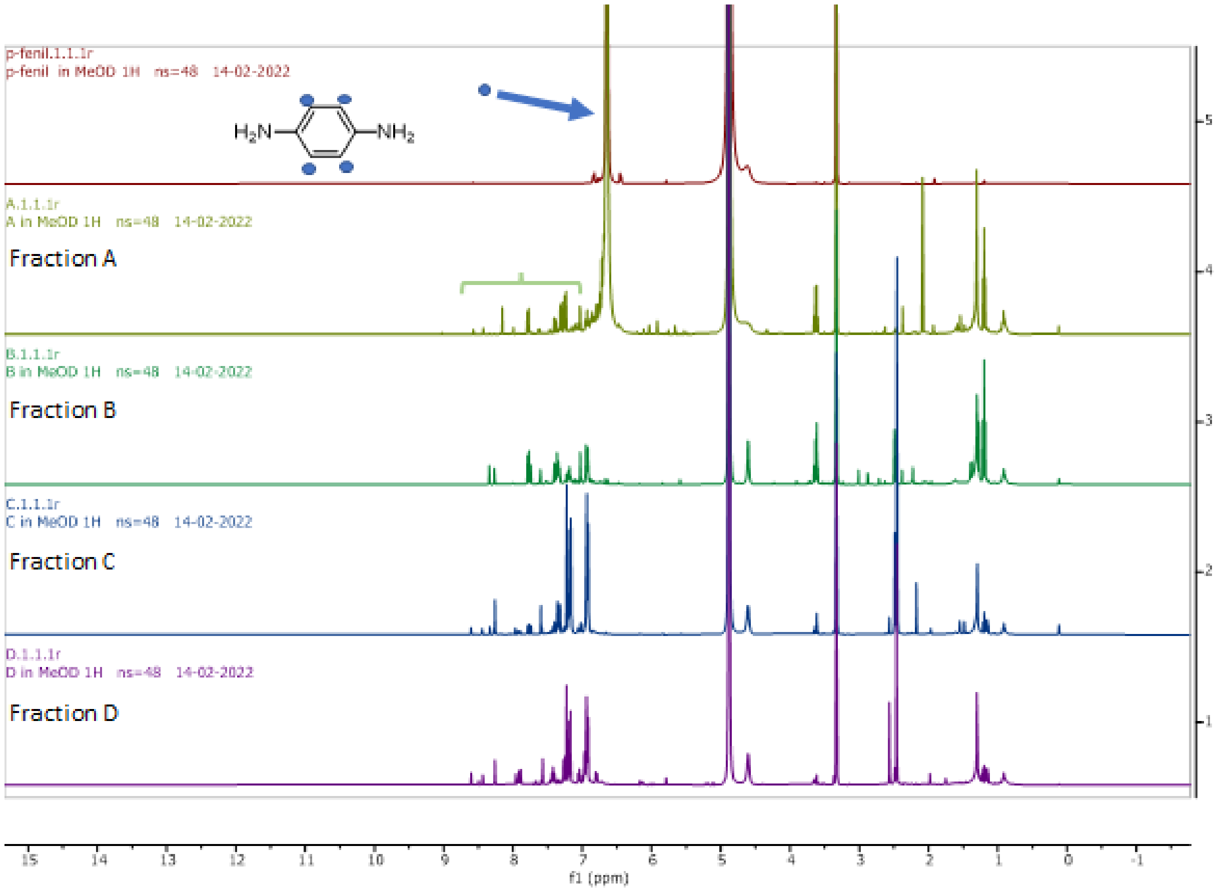

Figure S1 ^{1}H-NMR spectra of p-PDA and fractions A, B, C and D performed in MeOD.

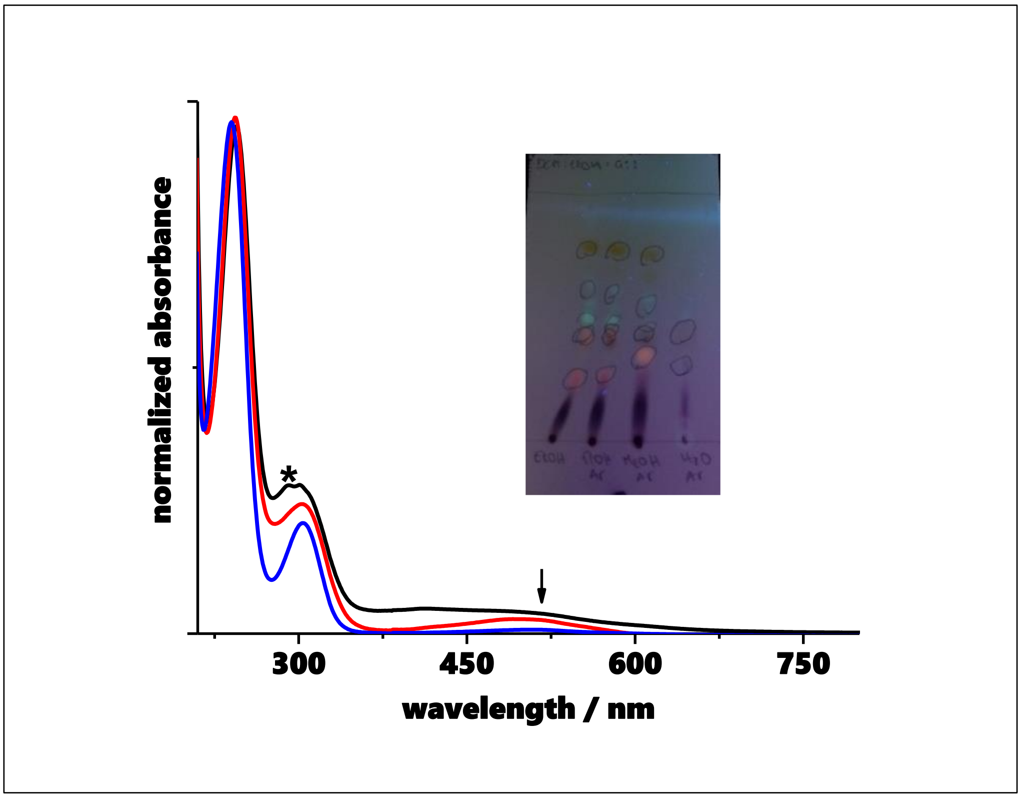

Figure S2 – UV-vis of CDs reaction mixtures obtained employing EtOH (black line), MeOH (red line) and water (blue line), respectively as solvents. Inset: TLC of the reaction mixtures obtained with the different solvents. Eluent: dichloromethane/EtOH 9:1. From left to right: EtOH, MeOH and water.

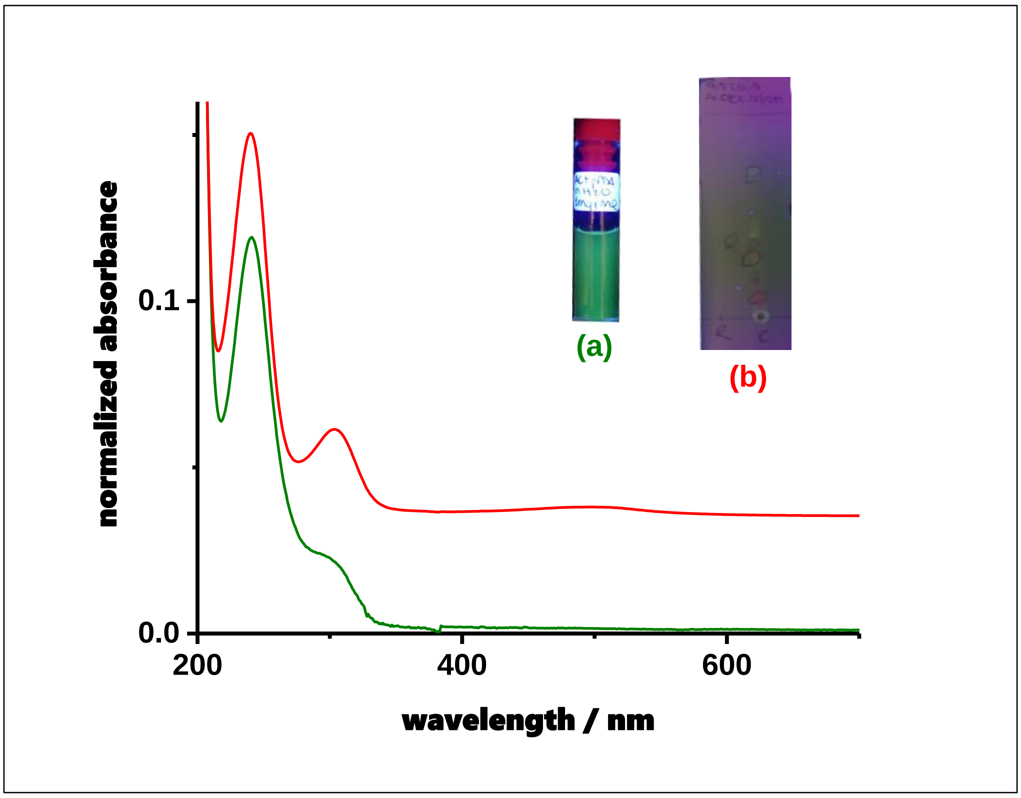

Figure S3 – UV-vis of CDs reaction mixtures in water obtained employing CA (green line) and urea (red line), respectively, as co-precursors. Inset: (a) Photograph of the reaction mixture in water obtained employing CA under 356 nm UV lamp; (b) TLC of the reaction mixture obtained with urea. Eluent: AcOEt/EtOH 9.5:0.5. From left to right: p-DPA and reaction mixture.
